# Allosteric inhibition of Aurora-A kinase by a synthetic vNAR domain

**DOI:** 10.1098/rsob.160089

**Published:** 2016-07-13

**Authors:** Selena G. Burgess, Arkadiusz Oleksy, Tommaso Cavazza, Mark W. Richards, Isabelle Vernos, David Matthews, Richard Bayliss

**Affiliations:** 1 Astbury Centre for Structural Molecular Biology, Faculty of Biological Sciences, University of Leeds, Leeds LS2 9JT, UK; 2 Department of Molecular and Cell Biology, University of Leicester, Leicester LE1 9HN, UK; 3 Centre for Therapeutics Discovery, MRC Technology, The Accelerator Building, Stevenage, Bioscience Catalyst, Gunnels Wood Road, Stevenage, Hertfordshire SG1 2FX, UK; 4 Cell and Developmental Biology program, Centre for Genomic Regulation (CRG) and UPF, Dr Aiguader 88, 08003 Barcelona, Spain; 5 Institució Catalana de Recerca i Estudis Avançats (ICREA), Pg. Lluis Companys 23, 08010 Barcelona, Spain

**Keywords:** antibody-assisted drug discovery, structural biology, biochemistry, protein kinase

## Abstract

The vast majority of clinically approved protein kinase inhibitors target the ATP-binding pocket directly. Consequently, many inhibitors have broad selectivity profiles and most have significant off-target effects. Allosteric inhibitors are generally more selective, but are difficult to identify because allosteric binding sites are often unknown or poorly characterized. Aurora-A is activated through binding of TPX2 to an allosteric site on the kinase catalytic domain, and this knowledge could be exploited to generate an inhibitor. Here, we generated an allosteric inhibitor of Aurora-A kinase based on a synthetic, vNAR single domain scaffold, vNAR-D01. Biochemical studies and a crystal structure of the Aurora-A/vNAR-D01 complex show that the vNAR domain overlaps with the TPX2 binding site. In contrast with the binding of TPX2, which stabilizes an active conformation of the kinase, binding of the vNAR domain stabilizes an inactive conformation, in which the αC-helix is distorted, the canonical Lys-Glu salt bridge is broken and the regulatory (R-) spine is disrupted by an additional hydrophobic side chain from the activation loop. These studies illustrate how single domain antibodies can be used to characterize the regulatory mechanisms of kinases and provide a rational basis for structure-guided design of allosteric Aurora-A kinase inhibitors.

## 1. Introduction

Kinase mutations drive many cancers through deregulated activity, and inhibitors of these kinases have revolutionized cancer treatment. Most current kinase inhibitors target the ATP-binding site, which is relatively straightforward to block with small molecules [1]. However, this site is also highly conserved among all protein kinases, and therefore many inhibitors have off-target effects. Patients treated with kinase inhibitors inevitably relapse due to drug resistance mechanisms such as kinase overexpression, mutation or the activation of bypass pathways, usually involving other kinases. Complex tumour biology, including genetic heterogeneity and drug resistance, may require the inhibition of more than one kinase for effective therapy [2–4]. Combinations of kinase inhibitors might address these issues, but combining these drugs safely and effectively is a challenge. This is thought to be, at least to some extent, due to the unfocused selectivity profiles of ATP-competitive inhibitors. Kinase inhibitors that bind to allosteric sites are more selective than ATP-competitive inhibitors [5]. However, allosteric inhibitors are more challenging to develop because kinase targets do not always have a clearly suitable allosteric site, and approaches to targeting such sites through screening and synthetic chemistry are less well developed than for the ATP-binding site. As a consequence, there are few examples of allosteric kinase inhibitors in the clinic. More recently, there have been several rational approaches to the development of allosteric inhibitors, which require foreknowledge of suitable binding sites [6–8].

Aurora-A is a Ser/Thr protein kinase that functions primarily in cell division and is overexpressed in breast, colon and other cancers [9]. Aurora-A interacts with and stabilizes N-Myc and c-Myc, transcription factors that underpin cancer development [10,11]. Consequently, ATP-competitive inhibitors of Aurora-A are under investigation in the treatment of neuroblastoma, a childhood cancer of the nervous system that is largely driven by N-Myc [12]. The activity of Aurora-A is stimulated by autophosphorylation of Thr288 in a flexible region termed the activation loop [13]. Aurora-A autophosphorylation is inefficient, but is stimulated by TPX2, a microtubule-associated protein that binds to the catalytic domain and stabilizes the kinase in an active conformation [14–17]. TPX2 binds to Aurora-A at two sites, stabilizing the positions of the αC-helix and activation loop through a structural mechanism that resembles the function of N- and C-terminal extensions present in AGC family kinases such as PKA [17]. Aurora-A is dysregulated in cancers and has been a popular target for drug discovery [9,18]. The first ATP-competitive inhibitors generated were equally effective against Aurora-B, but there are now a few compounds selective for Aurora-A that have undergone clinical trials, such as MLN8054 and MLN8237 [19,20]. To our knowledge, allosteric inhibitors of Aurora-A have not yet been identified and there has been no clearly described plan for their development. One potential approach to allosteric Aurora-A inhibitors would be to block the interaction with TPX2. However, a small molecule or peptide that mimics the binding of TPX2 to Aurora-A would be expected to stabilize the active conformation through the same mechanism as TPX2. A more effective strategy might be to develop inhibitors that stabilize an inactive conformation of Aurora-A.

In addition to conventional antibodies, camelids and cartilaginous fish (e.g. sharks, rays and skates) have antibodies that consist of a homodimer of two heavy chains [21–23]. For example, the immunoglobin new antigen receptor (IgNAR) from sharks consists of a heavy chain comprising five constant (C) domains and a single, variable domain, termed V or vNAR that binds targets. vNAR domains have an Ig fold consisting of only eight β-strands, in which the CDR2 region of a conventional VH domain is replaced by a short β-strand HV2 [24,25]. Camelid heavy chain antibodies also recognize epitopes through a single variable VHH domain. Distinct from the equivalent VH domain of conventional antibodies, the variable domains of heavy chain antibodies are independently stable and retain high affinity for epitope [23]. Single domain antibodies, also known as nanobodies, have become popular tools to stabilize proteins and thus facilitate their crystallization, and probe cryptic epitope sites [26–29]. Single domain antibodies have also been used as biotechnological tools to improve the pharmacokinetic properties of therapeutic fusion partners [30].

Here, we describe the identification of a vNAR single domain antibody based on a shark heavy chain antibody scaffold that binds and inhibits Aurora-A. The crystal structure of the complex indicates an allosteric mode of action that is antagonistic to the mechanism by which TPX2 activates the kinase. These studies provide a rational basis for structure-guided design of allosteric Aurora-A kinase inhibitors.

## 2. Results

### 2.1. Identification of a single domain antibody that inhibits Aurora-A

**Figure 1.**
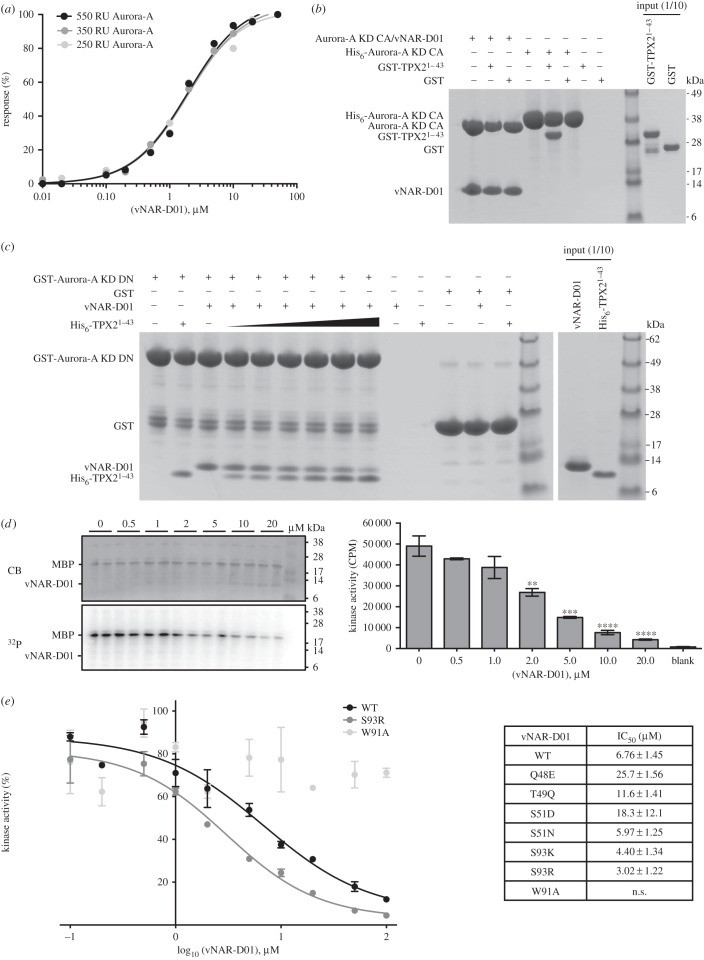
vNAR-D01 is an Aurora-A inhibitor that competes with TPX2. (*a*) Surface plasmon resonance binding assays between Aurora-A KD CA-Avi and vNAR-D01. The kinase was immobilized on Biacore Sensor SA chips at 550, 350 and 250 RU and interacted with 0.01–50 µM vNAR-D01. Maximum responses were plotted against vNAR-D01 concentration and fitted to a one-site specific binding equation (solid lines) in Prism6 (GraphPad) to calculate binding affinities. (*b*) Co-precipitation assay between the Aurora-A KD CA/vNAR-D01 complex or His_6_-Aurora-A KD CA and GST-TPX2^1–43^. The complex and Aurora-A were immobilized on Nickel Sepharose beads using the His_6_-tag on the vNAR domain and kinase, respectively. GST was used as a binding control. (*c*) Co-precipitation assay between GST-Aurora-A KD DN and vNAR-D01 and His_6_-TPX2^1–43^. In total, 2 µM GST-Aurora-A KD DN was immobilized on Glutathione Sepharose 4B beads and incubated with 5 µM vNAR-D01 and 0, 1, 2, 5, 10, 20 and 50 µM His_6_-TPX2 (black triangle). GST was used as a binding control. (*d*) *In vitro* kinase activity assay of Aurora-A KD in the presence of vNAR-D01. MBP was used as a generic kinase substrate. Reactions were analysed by SDS-PAGE (top left panel) and incorporation of radioisotope resolved by autoradiography (bottom left panel). Incorporation of radioisotope was measured by scintillation counting (right). Error bars represent the standard error for two independent reactions. ** = *p* < 0.01, *** = *p* < 0.001 and **** = *p* < 0.0001 using one-way ANOVA with Dunnett's post hoc test compared with the kinase only reaction. (*e*) *In vitro* kinase activity curves of Aurora-A KD in the presence of WT and mutant vNAR-D01 proteins. The kinase activity of Aurora-A KD was measured by the incorporation of radioisotope into the generic kinase substrate, MBP by scintillation counting in the presence of 0.1, 0.2, 0.5, 1, 2, 5, 10, 20, 50 and 100 µM vNAR-D01. Data were normalized to % kinase activity using the Aurora-A KD only reaction as 100% and plotted against vNAR-D01 concentration (right). Data were fitted to a log(inhibitor) versus response—variable slope in Prism6 (GraphPad) to calculate IC50s (right, solid line). n.s. = no significant inhibition observed.

A synthetic library of vNAR domains based on a scaffold isolated from Wobbegong shark was screened for binding to the kinase domain (KD) of human wild-type (WT) Aurora-A. All of the confirmed hits had the same amino acid sequence (electronic supplementary material, figure S1A). This protein, which we called vNAR-D01, was expressed in the periplasm of *Escherichia coli* with a non-cleavable C-terminal His_6_-tag, and purified using affinity and size exclusion chromatography (SEC). vNAR-D01 was verified to bind Aurora-A by far western blotting and SEC (electronic supplementary material, figures S1*b* and S2). Binding was not dependent on the phosphorylation status of the kinase (electronic supplementary material, figure S1*b*). The affinity of the interaction was determined to be 2 µM by surface plasmon resonance (SPR) (figure 1*a*; electronic supplementary material, figure S3*a*).

We investigated whether vNAR-D01 affected the interaction of Aurora-A with TPX2 using nickel sepharose to precipitate protein complexes through association with the His_6_-tag attached to a single component (figure 1*b*). Aurora-A KD C290A, C393A (CA) was used as this mutant routinely generates crystals of higher resolution than the WT KD [31]. Here, untagged Aurora-A KD CA was efficiently co-precipitated by vNAR-D01, but GST-TPX2^1–43^ did not co-precipitate with the complex. By contrast, GST-TPX2^1–43^ was efficiently precipitated by His_6_-tagged Aurora-A KD CA in the absence of the vNAR domain. This suggested competition between TPX2 and vNAR-D01 for Aurora-A binding. His_6_-TPX2^1–43^ or vNAR-D01 robustly co-precipitated with GST-Aurora-A KD D274N (DN; the mutation results in a catalytically inactive form of the kinase) immobilized on Glutathione Sepharose beads, but competition was established through a dose-dependent decrease in vNAR-D01 binding as the concentration of TPX2 was increased (figure 1*c*). We used *Xenopus* egg extracts to investigate the competition between vNAR-D01 and Aurora-A in a situation closer to the physiological pathway (electronic supplementary material, figure S3*b*). In this system, TPX2 and Aurora-A robustly interact when extracts are supplemented with RanGTP [14]. However, co-precipitation of vNAR-D01 with *Xenopus* Aurora-A was not observed and we concluded that we would require a vNAR domain of higher binding affinity and/or generated against the *Xenopus* protein to warrant further investigation.

In light of the competition between vNAR-D01 and the Aurora-A activator, TPX2^1–43^, we asked whether vNAR-D01 might also activate the kinase. vNAR-D01 was added to kinase assays based on incorporation of ^32^P into a substrate protein to quantify the kinase activity of Aurora-A (figure 1*d*,*e*). vNAR-D01 showed a dose-dependent inhibition of Aurora-A, an effect opposite to that of TPX2. To address the mechanism of Aurora-A inhibition by vNAR-D01, we crystallized the complex in two different forms and determined the structures by X-ray crystallography to limiting resolutions of 1.67 Å and 1.79 Å, respectively (electronic supplementary material, table S1). In both crystal forms the asymmetric unit contained a single complex of Aurora-A KD CA/vNAR-D01 with a 1 : 1 stoichiometry. The two crystal structures are very similar, with an overall Cα RMSD of 0.97 Å (electronic supplementary material, figure S4). ADP is bound in the ATP-binding pocket but there are no magnesium ions present in the active site of either structure, and the side chain of Asp274, which coordinates magnesium ions in other Aurora-A structures, is displaced from the active site and instead interacts with the side chain of His254.

### 2.2. The binding sites of vNAR-D01 and TPX2 on Aurora-A overlap

**Figure 2.**
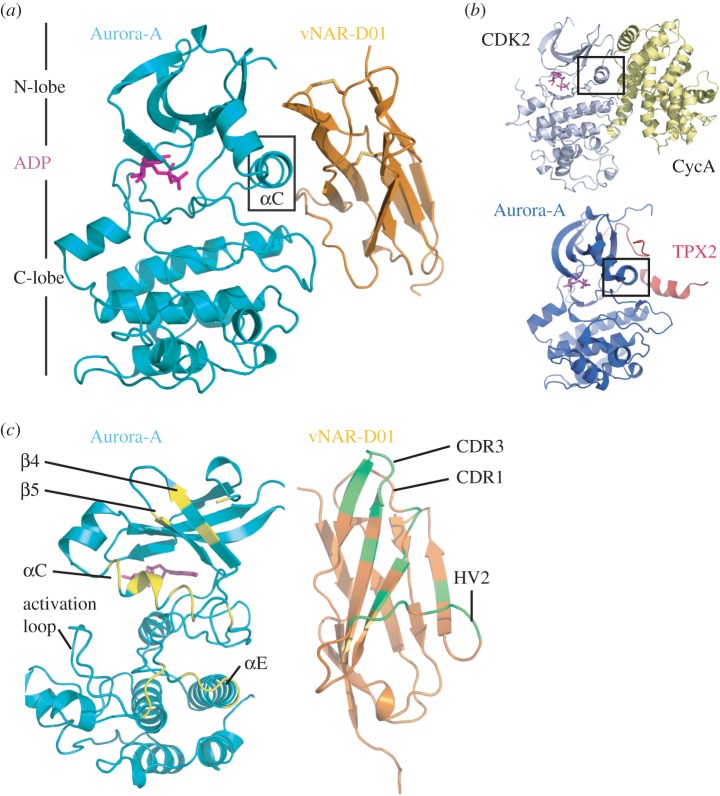
Crystal structure of Aurora-A/vNAR-D01 complex. (*a*) Cartoon representation of the complex structure (crystal form 1). Aurora-A is coloured teal and vNAR-D01 is coloured orange. (*b*) Structures of CDK2/Cyclin-A (PDB 1FIN) and Aurora-A/TPX2 (PDB 1OL5) complexes. The αC-helix is marked with a black rectangle in panels (*a*) and (*b*). (*c*) The interacting regions of the Aurora-A and vNAR-D01 are shown as contrasting colours on the individual proteins (yellow and green, respectively).

In the crystal structure of the complex, Aurora-A adopts a canonical kinase fold, with a molecule of ADP sandwiched between the N- and C-lobe (figure 2*a*). vNAR-D01 has an Ig fold with two disulfide bridges. vNAR-D01 makes contacts with both lobes of Aurora-A, but does not closely approach the ATP-binding pocket. The interface is centred on the αC-helix within the N-lobe of Aurora-A. This helix within the kinase fold bears residues that are critical for catalysis and is often the site of interactions with regulatory binding partners as exemplified by the complex of Cyclin-A/CDK2 and TPX2/Aurora-A (figure 2*b*).

**Figure 3.**
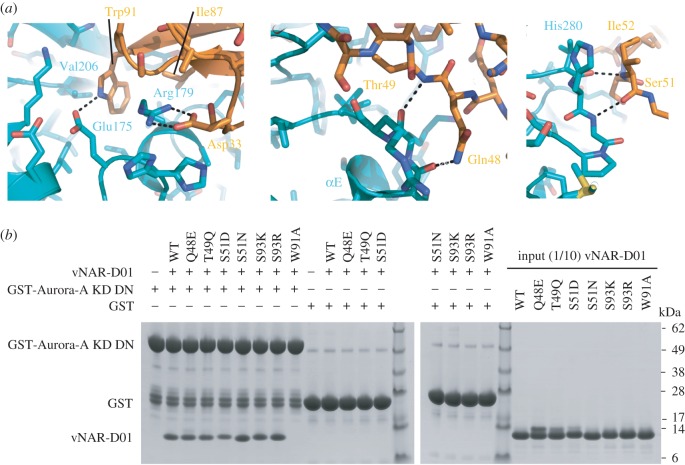
Details of the molecular recognition in the Aurora-A/vNAR-D01 complex. (*a*) Key interactions are shown in the three panels. Aurora-A is coloured teal and vNAR-D01 is coloured orange. (*b*) Co-precipitation assay between GST-Aurora-A KD DN and WT, and mutant vNAR-D01 constructs. GST-Aurora-A KD DN was immobilized on Glutathione Sepharose 4B beads and then incubated with vNAR-D01 proteins. GST was used as a binding control.

The interface of the complex buries 950 Å^2^ of surface molecule per molecule of vNAR-D01 or Aurora-A. The binding footprint of vNAR-D01 on Aurora-A comprises regions of the αC-helix, β4 strand, activation loop and the N-terminus of helix αE (figure 2*c*). All three variable regions of vNAR-D01 contact the kinase surface: Asp33 from CDR1 forms a salt bridge with Arg179 (Aurora-A αC); residues 48–49 of HV2 contact the N-terminus of αE; Ser51 (main chain only) and Ile52 (main chain and side chain) of HV2 interact with the activation loop sequence Val279-His280-Ala281; the side chains of CDR3 residues Ile87 and Trp91 insert into a hydrophobic pocket formed between αC and β4; and the Trp91 side chain makes an H-bond with the Glu175 side chain within this pocket (figure 3*a*). In addition, Asn36 and Tyr38 from βC interact with the αC-helix through an H-bond with the side chain of Glu183 (and van der Waals contact with the side chain of His187). These interactions are mostly conserved between the two crystal forms of the complex, with the exception of the contacts between HV2 and activation loop (electronic supplementary material, figure S4*d*). The binding conformation of vNAR-D01 is probably stabilized by a non-canonical disulfide bond between Cys30 of CDR1 and Cys90 CDR3, a characteristic feature of type II/III vNAR domains (figure 2*a*; electronic supplementary material, figure S1A) [32].

To validate the crystal structure, we generated a point mutation in vNAR-D01, W91A, designed to disrupt the interaction. We also generated a series of mutations with the aim of enhancing the interaction: Q48E, T49Q, S51D, S51N, S93 K and S93R. All mutant vNAR-D01 proteins were expressed and purified, and tested for binding to Aurora-A by co-precipitation assay (figure 3*b*). As predicted, the W91A mutation completely disrupted the interaction and the other mutants retained binding. The circular dichroism (CD) spectrum of WT vNAR-D01 and the W91A mutant were similar, indicating no changes in secondary structure resulted from mutation (electronic supplementary material, figure S5).

We then determined the potency of Aurora-A inhibition by vNAR-D01 and the point mutants using a kinase assay in which vNAR-D01 inhibited Aurora-A with a IC_50_ of 6.76 µM (figure 1*e*; electronic supplementary material, figure S6*a*). We could not detect any inhibition of Aurora-A by the W91A mutant vNAR-D01, consistent with the loss of interaction. Three of the other mutants exhibited less potent inhibition: Q48E, T49Q and S51D. Three of the mutants exhibited more potent inhibition: S51N, S93 K and S93R. The most potent inhibitor was S93R-vNAR-D01, with an IC_50_ of 3.02 µM (figure 1*e*; electronic supplementary material, figure S6*a*). A crystal structure of S93R-vNAR-D01 showed only a minor change in conformation from vNAR-D01: the side chain of Arg93 forms an intramolecular salt bridge with Glu95 (electronic supplementary material, figure S6*b*).

### 2.3. vNAR-D01 destabilizes the αC-helix of Aurora-A

**Figure 4.**
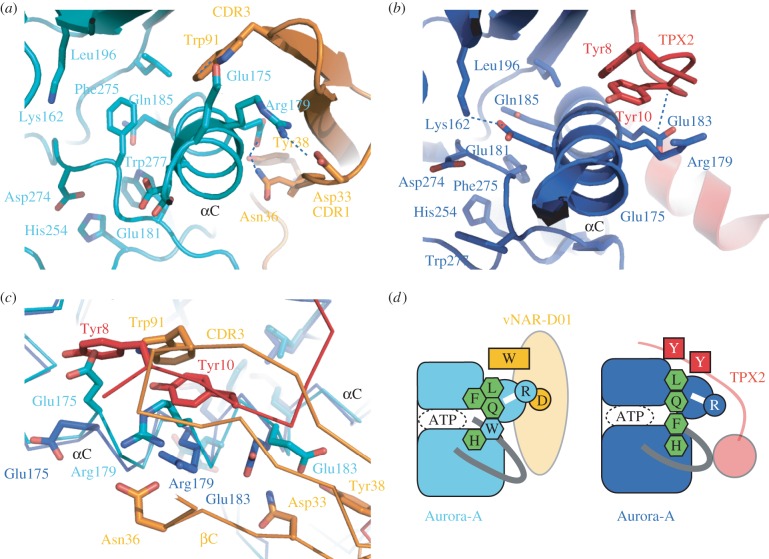
The mechanism of allosteric inhibition of Aurora-A by vNAR-D01 is antagonistic to TPX2 activation. (*a*) Aurora-A/vNAR-D01 complex viewed along the αC-helix. (*b*) Aurora-A/TPX2 complex, equivalent view to that shown in (*a*). (*c*) Superposed structures of Aurora-A/vNAR-D01 (teal/orange) and Aurora-A/TPX2 (blue/red) complexes viewed with the αC-helix running from left to right. Note that the binding site of the CDR3 loop of vNAR-D01 on Aurora-A overlaps with the binding site of TPX2 residues Tyr8 and Tyr10. (*d*) Schematic of the structural basis by which vNAR-D01 and TPX2 stabilize distinct conformations of Aurora-A through binding at the same site. Key residues are shown as single-letter notation. Canonical R-spine residues are shown as green hexagons and the additional residue that joins the R-spine in inactive Aurora-A is shown as a light blue hexagon.

The conformation of Aurora-A in complex with vNAR-D01 lacks key hallmarks of an active kinase: there is no Lys-Glu salt bridge and the hydrophobic R-spine is incorrectly formed (figure 4*a*). In its active conformation, when bound to TPX2, the kinase has a salt bridge between Lys162 and Glu181, and the R-spine is correctly assembled by the interactions between the side chains of Leu196, Gln185, Phe275 and His254 (figure 4*b*) [17,33,34]. Indeed, the R-spine of Aurora-A in complex with vNAR-D01 is disrupted by the presence of an additional side chain: Trp277, which is predicted to interact with substrate peptide in the active kinase, is twisted inwards and fills the space that Phe275 (of the DFG-motif) would normally occupy. Phe275 is displaced to a position between Lys162 and Glu181. Trp277 also H-bonds with Gln185, twisting this R-spine component out of position. These conformational changes are coupled to a distortion of the αC-helix in the vicinity of Glu181. The distortion is stabilized by interactions on all sides: the side closest to β4 is stabilized by Trp91 of vNAR-D01 CDR3; the side facing out to solution is stabilized by Asp33, Asn36 and Tyr38 of vNAR-D01 CDR1 and strand βC; and the inside is stabilized by conformational changes in the activation loop, most notably Phe275 and Trp277. The position of the αC-helix is incompatible with TPX2 binding, as side chains of Glu175 and Arg179 would clash with Tyr8 and Tyr10 (figure 4*c*). In summary, TPX2 stabilizes the active conformation of Aurora-A and vNAR-D01 stabilizes an inactive conformation (a schematic overview is shown in figure 4*d*).

**Figure 5.**
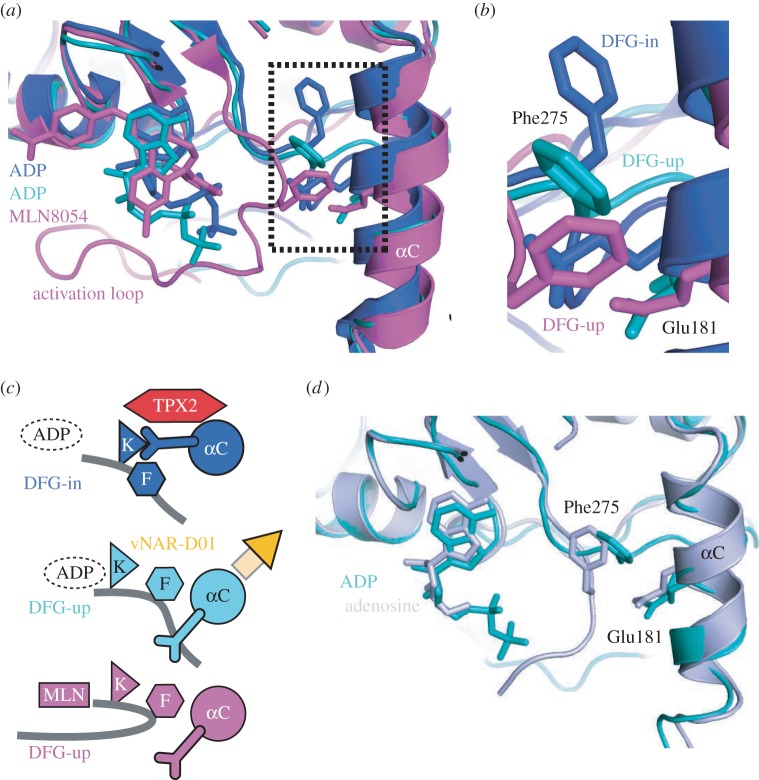
Aurora-A in complex with vNAR-D01 adopts a DFG-up conformation. (*a*) Superposed structures of Aurora-A in complex with vNAR-D01/ADP (teal), TPX2/ADP (dark blue; PDB 1OL5) and MLN8054 (magenta; PDB 2WTV). (*b*) Magnified view of Phe275 and Glu181. (*c*) Schematic to show how the DFG-up conformation disrupts the Lys-Glu salt bridge. The activation loop is shown as a grey line, with the position of Phe275 marked with a hexagon labelled ‘F’. In the active conformation of Aurora-A (dark blue, top image), a salt bridge is formed between Lys162 (marked with a triangle labelled ‘K’) and Glu181 (shown as a Y-shaped appendage on the αC-helix). Distortion of the αC-helix by vNAR-D01 (orange, central image) breaks the Lys-Glu salt bridge and creates a hydrophobic pocket for Phe275. A similar configuration of the αC-helix and Phe275 is observed in the structure of Aurora-A bound to MLN8054 (magenta, lower image), which induces a rearrangement of the activation loop. (*d*) Superposed structures of Aurora-A in complex with vNAR-D01/ADP (teal) and adenosine (lilac).

The conformation of Aurora-A bound to vNAR-D01 bears a striking resemblance to Aurora-A in complex with MLN8054, an ATP-competitive inhibitor that induces conformational changes in the catalytic domain (figure 5*a*) [19,35]. MLN8054 interacts extensively with the DFG-motif and causes a substantial conformational change in the activation loop. MLN8054 and vNAR-D01 both stabilize a distortion in the αC-helix that disrupts the Lys-Glu salt bridge by moving the side chain of Glu181 away from the ATP-binding site. The distortion of the αC-helix is coupled to a shift in the position of the side chain of Phe275, which fills the space vacated by Glu181 (figure 5*b*,*c*). Therefore, the mechanism by which vNAR-D01 inhibits Aurora-A recapitulates some of the features of MLN8054, without blocking the binding of ATP. Furthermore, the conformation of Aurora-A trapped by vNAR-D01 is also similar to that observed for Aurora-A in complex with adenosine (figure 5*d*) [36]. Thus, three structures of Aurora-A exhibit a distorted αC-helix coupled to changes in the position of Phe275, which represent three distinct complexes of the kinase crystallized under three different conditions. It therefore seems likely that the conserved features of these structures represent a physiologically relevant conformation of Aurora-A, one of many that may exist when the kinase is in a dynamic state, which can be captured by ligands or vNAR-D01.

## 3. Discussion

### 3.1. Single domain antibodies as tools to manipulate kinase structure and activity

One attraction of targeting allosteric sites is that they are more or less unique to an individual kinase, and therefore allosteric inhibitors are potentially more selective than ATP-competitive inhibitors. Among human protein kinases, the three residues that contact Trp91 of vNAR-D01 (Asp175, Arg179 and Val206) are identical only in Aurora-C, and only Aurora-B has a single conservative substitution (Ile in place of Val206). All other human protein kinases have at least one non-conserved residue, and of these, the pairing of an acidic residue/basic residue at the positions equivalent to Asp175 and Arg179 is found only in kinase interacting with stathmin (KIS) [37]. vNAR-D01 is likely to be specific to the Aurora kinase family, with the possible exception of KIS. However, we have not yet carried out a biochemical study of the specificity of vNAR-D01, which would be necessary to support this bioinformatical analysis.

Previous work showed that single domain antibodies could be used to trap a kinase in a specific conformation and modulate kinase activity through binding to a regulatory domain [38,39]. Structures of cyclin-G associated kinase (GAK) in two different conformations were captured by co-crystallization with two nanobodies (NbGAK_1 and NbGAK_4), which bind to two different regions of the kinase surface, both of which are distinct from those we observed for vNAR-D01 (electronic supplementary material, figure S7) [38]. In complex with NbGAK_1, GAK was monomeric with an ordered activation loop, whereas NbGAK_4 trapped a dimeric configuration with disordered activation loops. Despite the differences observed in the crystal structures, the nanobodies had very little effect on GAK activity in solution: NbGAK_4 modestly enhanced kinase activity, while NbGAK_1 had no effect. *Toxoplasma gondii* Calcium-dependent protein kinase 1 (*Tg*CDPK1) consists of a catalytic domain and a regulatory domain that either inhibits or promotes kinase activity in response to calcium. A single domain VHH antibody that interacts with the regulatory domain of *Tg*CDPK1 (1B7) inhibits the kinase by stabilizing the regulatory domain in the inhibitory conformation [39]. These two studies illustrated the potential of nanobodies as tools for biochemical and structural analysis of kinases. Here, we show that the single domain antibody vNAR-D01 binds directly to the catalytic domain of Aurora-A and inhibits kinase activity through an allosteric mechanism. Taken together, these studies demonstrate the versatility of single domain antibodies as molecular probes to investigate kinase regulatory mechanisms. However, vNAR-D01 does not bind Aurora-A with sufficient affinity to be a useful tool in cell-based assays, which may require a KD of less than 100 nM, and so we will affinity mature this scaffold to improve its potency. In addition, to avoid any potential folding issues of the inhibitor in a reducing cellular environment, we will screen alternative, non-antibody scaffolds that lack disulfide bonds.

### 3.2. Allosteric activators and inhibitors

vNAR-D01 inserts Trp91 into a hydrophobic pocket formed by the αC-helix and strand β4 of Aurora-A. This pocket plays an important role in the regulation of Aurora-A by TPX2, and more generally in the regulation of AGC and related kinases, through binding of a peptide bearing a hydrophobic motif [40]. These interactions can be in *cis,* as in PKA, or in *trans*, as in Aurora-A, or PDK1, and activate the kinase through stabilization of an active conformation [41]. Specifically, the interaction of TPX2 stabilizes the αC-helix of Aurora-A to ensure that the Lys-Glu salt bridge is formed (figure 5). This regulatory, hydrophobic pocket presents an attractive target for the rational development of allosteric kinase inhibitors. Indeed, the equivalent hydrophobic (PIF) pocket of PDK1 has successfully been targeted by small molecules, both directly and by tethering approaches [6–8]. Here, we present an orthogonal approach to targeting this pocket via a single domain antibody. Although compounds based on the structure of Aurora-A in complex with TPX2 would be expected to activate the kinase, the Aurora-A/vNAR-D01 structure could form the basis for rational design of allosteric inhibitors. More generally, we believe that single domain antibodies will be useful to trap kinases in an inactive conformation to facilitate the development of allosteric kinase inhibitors, and will synergize with other small molecule, peptide and computational approaches.

## 4. Material and methods

### 4.1. Cloning, protein expression and purification

The vectors pETM11 Aurora-A KD, pET30TEV Aurora-A KD CA, pGEX-cs Aurora-A KD DN, pGEX-cs TPX2^1–43^ and pET30TEV TPX2^1–43^ were produced in earlier work [17,42,43]. The vector pGEX-2T was used for the expression of GST. The expression vector for Aurora-A KD-Avi was produced by sub-cloning of the gene for Aurora-A KD into pETM6T1 for expression with an N-terminal TEV cleavable His-NusA tag. A C-terminal non-cleavable Avi-tag was added to the coding sequencing of Aurora-A KD by primer extension PCR.

GST, TPX2^1–43^ and Aurora-A KD, KD CA and KD DN were expressed and purified as stated in earlier work [17,43]. The expression vector for Aurora-A KD-Avi was co-transformed into *E. coli* B834 cells with the vector pBirAcm for co-expression with biotin ligase and cultured as recommended by the supplier (Avidity LLC, USA). His-NusA Aurora-A KD-Avi was purified by immobilized metal ion affinity chromatography (IMAC) using a HiTrap Chelating Sepharose HP column (GE Healthcare) as per the manufacturer's instructions. The His-NusA tag was removed by overnight TEV cleavage. IMAC was repeated to remove the TEV protease, expression tag and biotin ligase. Q-Sepharose chromatography (GE Healthcare) was performed according to the manufacturer's instructions to improve protein purity. As a final polishing step, Aurora-A KD-Avi was subject to SEC on a HiLoad 16/600 Superdex 200 column (GE Healthcare) equilibrated in 20 mM Tris pH 7.0, 200 mM NaCl, 5 mM MgCl_2_, 5 mM β-mercaptoethanol and 10% glycerol. Biotinylation of purified Aurora-A KD-Avi was confirmed by western blotting with an anti-biotin primary antibody (abcam, 1 : 5000, ab53494).

Expression vectors for vNAR constructs were transformed into CodonPlus RIL *E. coli* cells and grown in LB media at 37°C until an induction OD_600_ approximately 0.6 was attained and 0.6 mM IPTG added. Cultures were incubated overnight at 21°C prior to cell harvesting by centrifugation. Protein purification was performed as described in other work for His-tagged constructs [44]. The protein was subject to a final SEC step as described for Aurora-A KD-Avi.

### 4.2. Crystal structure determination

To make the Aurora-A KD CA/vNAR-D01 complex, the proteins were mixed at a stoichiometry of 1 : 1.2, respectively, and were subject to SEC. Fractions containing complex were combined and concentrated to 16.5 mg ml^−1^, and incubated with 5 mM ADP/MgCl_2_ for 1 h prior to crystallization screening trials. Screens were set up in 96-well sitting drop MRC plates using a mosquito LCP crystallization robot (ttplabtech) and incubated at 295 K. Crystals were observed after 2 days of incubation in a number of conditions. Further hits were identified in the following three weeks. Diffraction data were collected at Diamond Light Source (Oxford, UK) on beamline IO4-1. Two different space groups were observed and the data from the highest resolution crystals were processed for structure determination. Data used were from a single crystal. Crystal form 1 was produced using 0.2 M ammonium sulfate, 0.1 M sodium acetate pH 4.6, 12.5% PEG 4000 as the mother liquor. Crystal form 2 was grown using 0.2 M ammonium sulfate, 0.1 M bis-Tris pH 5.5, 25% PEG 3350 as a precipitant. Both crystals were cryoprotected by the addition of 30% ethylene glycol and flash-cooled. Data processing was carried out using the ‘-3daii’ mode on the *xia2* automated data reduction platform available at Diamond Light Source. The structure of Aurora-A KD CA/vNAR-D01 was solved by molecular replacement using the structure of Aurora-A KD CA (PDB entry 4CEG) [31] and the structure of a Spotted Wobbegong shark vNAR domain (PDB 2COQ) [45] as a model. The structure was solved and rigid body refined with Phenix. Model building was carried out with Coot. MolProbity was used for Ramachandran analysis.

Crystals of Aurora-A KD CA/vNAR-D01 S93R were produced as for the WT complex using 0.1 M citric acid pH 5.0, 20% PEG 6000 as a precipitant and diffraction data were collected at Diamond Light Source on beamline IO4-1. Structure determination was carried out as described above with data from a single crystal cryoprotected in 30% ethylene glycol using the WT complex crystal form 2 as a model.

### 4.3. Co-precipitation assays

For co-precipitation assays, 100 µg bait protein was immobilized on 20 µl resin equilibrated in assay buffer. The resin was pelleted by centrifugation and washed twice with assay buffer. The beads were resuspended in assay buffer to which 100 µg prey protein was added and incubated for a further 2 h at 4°C. The reactions were washed twice with assay buffer prior to the addition of 20 µl SDS-loading buffer and SDS-PAGE analysis. Glutathione Sepharose 4B beads (GE Healthcare) equilibrated in the assay buffer, 50 mM Tris pH 7.5, 150 mM NaCl, 5 mM MgCl_2_, 5 mM β-mercaptoethanol and 0.02% TWEEN 20 were used in assays where GST-Aurora-A KD DN and GST were used as bait. Nickel Sepharose resin (GE Healthcare) equilibrated in the assay buffer, 50 mM Tris pH 7.5, 150 mM NaCl, 40 mM imidazole and 0.1% TWEEN-20 were used to immobilize His_6_-Aurora-A KD CA and vNAR-D01 as bait proteins.

For competition co-precipitation assays performed with a gradient of 0–50 µM His_6_-TPX2^1–43^ and 5 µM vNAR-D01, 2 µM GST-Aurora-A KD DN was immobilized on Glutathione Sepharose 4B beads and reactions were performed as described above.

### 4.4. *In vitro* kinase activity assays

Kinase assays were performed as stated in earlier work [43]. To determine the IC50 of vNAR-D01 constructs, kinase reactions were performed in the presence of 0–100 µM vNAR domain and analysed by scintillation counting. Data were normalized to % kinase activity using the Aurora-A KD only reaction as 100% and plotted against vNAR-D01 concentration. Data were fitted to a log(inhibitor) versus response—variable slope in Prism6 (GraphPad) to calculate the IC50.

### 4.5. Surface plasmon resonance

SPR assays were performed on a BIAcore 3000 instrument using running buffer, 10 mM Hepes pH 7.4, 150 mM NaCl, 5 mM MgCl_2_, 10% glycerol and 0.005% TWEEN-20. Aurora-A KD-Avi was immobilized on three flow-cells of BIAcore SA sensor chips (GE Healthcare) at three immobilization levels (250, 350 and 500 RU) and the fourth was left blank. vNAR-D01 was diluted into running buffer to a range of concentrations and injected over the chips at 40 µl min^−1^ for 375 s. Sensorgrams were recorded for each injection and processed using BIAevaluation 3.0 software (Biacore AB) and the data recorded in the blank flow-cell subtracted from each sensorgram. Equilibrium response at 350 s was plotted against concentration for each quantity of immobilized Aurora-A KD-Avi and fitted by nonlinear regression to a binding isotherm using Prism6.

## Supplementary Material

Characterisation of vNAR-D01. Characterization of the Aurora-A/vNAR-D01 complex by SEC. Characterization of the Aurora-A/vNAR-D01 interaction. Comparison of two crystal forms of Aurora-A KD CA/vNAR-D01. CD spectra of wild-type vNAR-D01 and the point mutant, W91A.

## Data accessibility

Protein complex structures: RCSB PDB codes 5L8 J, 5L8 K and 5L8 L.
