# Supplementary material for: Allosteric inhibition of Aurora-A kinase by a synthetic vNAR domain
Source: Open Biol. 2016 Jul 13;6(7):160089. doi: 10.1098/rsob.160089 (PMC4967828; doi:10.1098/rsob.160089)
Supplement: Characterisation of vNAR-D01. Characterization of the Aurora-A/vNAR-D01 complex by SEC. Characterization of the Aurora-A/vNAR-D01 interaction. Comparison of two crystal forms of Aurora-A KD CA/vNAR-D01. CD spectra of wild-type vNAR-D01 and the point mutant, W91A. [file rsob160089supp1.pdf]

Supplementary methods

Phage Display Bio-panning

A synthetic Wobbegong shark vNAR library cloned into a derivative of the phagemid vector, pUC119 was supplied by Cyclogenix Ltd (Aberdeen, UK). Phage rescue was carried out by infecting *E. coli* TG-1 cells transformed with the library at an OD_{600} ~0.5 with M13K07 helper phage (New England Biolabs) at a multiplicity of infection (MOI) of 20. After overnight growth at 30 °C, the bacteria were pelleted by centrifugation and the phage purified from the culture supernatant by 20% PEG/2.5 M NaCl precipitation on ice for 1 hour. The phage were resuspended in PBS prior to the addition of 20% glycerol and storage at -20 °C.

Aurora-A KD-Avi was used for bio-panning against the vNAR library. Three panning rounds were performed. For the first round, 50 nM antigen and 2 x 10^{12} phage particles in PBS containing 2% milk and 0.1% TWEEN-20 were incubated for 1 hour at room temperature (RT). This mixture was added to 100 µl Dynabeads M-280 Streptavidin (Invitrogen) pre-blocked in PBS with 4% milk and incubated for 15 minutes at RT prior to six washes with PBS plus 0.1% TWEEN-20 followed by two final washes with PBS only. Bound phage was eluted from the beads with triethylamine and neutralized with Trizma-HCl pH 8.0. Mid-log phase *E.coli* TG-1 cells were infected with the eluted phage and incubated at 37 °C for 1 hour prior to plating out on 2YT plus 2% glucose and 100 µg/ml ampicillin (2YTGA) bioassay agar plates. Dilutions were made to calculate output titres. Plates were incubated overnight at 30 °C. Cells were recovered from the bioassay plate in 2YTGA media plus 15% glycerol and stored at -80 °C. For the next round of panning, the library was rescued by inoculation of 50 ml 2YTGA media with 50 µl output glycerol stock and grown until an OD_{600} ~0.5 was attained. The culture was infected with a 10-fold excess of helper phage and incubated at 37 °C for 1 hour. The culture media was exchanged for 50 ml 2YT supplemented with 100 µg/ml ampicillin and 50 µg/ml kanamycin by centrifugation and incubated overnight at 30 °C. Bacteria were pelleted and the phage library recovered from the supernatant by 20% PEG/2.5 M NaCl precipitation. 100 µl of the phage library was used as the input for the next round of panning following the methods described above.

For the second round of bio-panning, the concentration of Aurora-A KD-Avi was lowered to 10 nM and non-specific binders to the resin were removed by pre-incubation of the library with pre-blocked Dynabeads M-280 Streptavidin beads for 1 hour. In the third round, the concentration of Aurora-A KD-Avi was reduced further to 1 nM. Individual clones were tested in a Streptavidin based phage-ELISA. Phagemids were isolated from positive clones and sent for DNA sequencing (GATC, Germany). The resulting vNAR construct named D01 was subcloned into pET26b(+) using the restriction sites (5'/3') NcoI/NotI in-frame with the PelB leader for recombinant expression in the periplasm of *E. coli*. Point mutations in the construct were introduced by site-directed mutagenesis.

Far Western Blotting Analysis

10 μM Aurora-A and vNAR-D01 were subject to SDS-PAGE analysis. Far Western blotting was performed using vNAR-D01 (1:1000, 1 mg/ml). The blot was incubated overnight with vNAR-D01 in Tris buffered saline plus 0.05% TWEEN-20 and 5% BSA. Binding of vNAR-D01 was detected by anti-His (1:5000, Clontech Cat no. 631212) Western blotting.

Experiments in Xenopus laevis egg extracts

Fresh cytostatic-factor-arrested *Xenopus* egg extracts (CSF extract) were prepared as previously described (1). CSF extract was supplemented with CSF-XB buffer for control, or with 5.3 or 25 μM of vNAR domain and incubated for 15 minutes at 20 °C before the addition of 15 μM RanGTP or CSF-XB buffer as control. After incubation for 20 minutes at 20 °C, the extract was placed on ice to perform the anti-AurA immunoprecipitation (IP).

For the AurA IPs, 24 μl protein A-conjugated Dynabeads 280 (Invitrogen) were washed three times with 1 ml of PBS–Triton (0.1%) and then incubated for 30 minutes at RT on a rotating wheel with 6 μg of anti-AurA antibody. Antibody-coated beads were washed twice with 1 ml of PBS–Triton (0.1%) and twice with 1 ml of CSF-XB. The beads were then resuspended with 60 μl of CSF extract containing the vNAR domain and RanGTP. The mixture was incubated on ice for 1 hour and the beads retrieved on a magnet for 10 minutes, washed twice with 0.5 ml of CSF-XB and twice with 0.5 ml of 0.1% PBS–Triton, both containing phosphatase inhibitors (100 mM NaF, 80 mM β-glycerophosphate and 1 mM Na_3VO_3). Proteins were eluted from the beads by incubation in SDS loading buffer for 10 min at RT and the supernatant used for SDS–PAGE and Western blot analysis.

Recombinant Proteins and Antibodies

RanGTP was purified and loaded with GTP as previously described (2). The rabbit polyclonal anti-TPX2 antibody was produced as described in (3). The rabbit polyclonal anti-AurA antibody was used as described in (4). Home-made antibodies were used at a final concentration of 1 μg/ml for Western blots. The mouse monoclonal anti-AurA ICI1 antibody was a gift from C. Prigent and was used at 1:200. The mouse monoclonal anti-His antibody (Qiagen ref 34660) was used at 1:1000.

CD spectroscopy

Wild-type and W91A vNAR-D01 were diluted to 1 mg/ml in 20 mM sodium phosphate pH 7.0. Spectra were collected on a Chirascan+ instrument (Applied Photophysics) using a 0.01 cm pathlength quartz cell at 21 °C and are shown as the average of three replicates after baseline subtraction.

References

1. Desai A, Murray A, Mitchison TJ, & Walczak CE (1999) The use of *Xenopus* egg extracts to study mitotic spindle assembly and function *in vitro*. *Methods Cell Biol* 61:385-412.

2. Brunet S*, et al.* (2004) Characterization of the TPX2 domains involved in microtubule nucleation and spindle assembly in *Xenopus* egg extracts. *Mol Biol Cell* 15(12):5318-5328.
3. Wittmann T, Wilm M, Karsenti E & Vernos, I (2000) TPX2, a novel *Xenopus* MAP involved in spindle pole organization. *J Cell Biol* 149(7):1405-1418.
4. Peset I*, et al.* (2005) Function and regulation of Maskin, a TACC family protein, in microtubule growth during mitosis. *J Cell Biol* 170(7):1057-1066.

A

MARVDQTPRIATKETGESLTINCVLRDTACALDSTNWYRT
KLGSTKEQTISIGGRYSETVDEGSNSASLTI RDLRVEDSG
TYKCKAIDSCWLSREGAGTVLTVKGGAAALEHHHHHH-

B

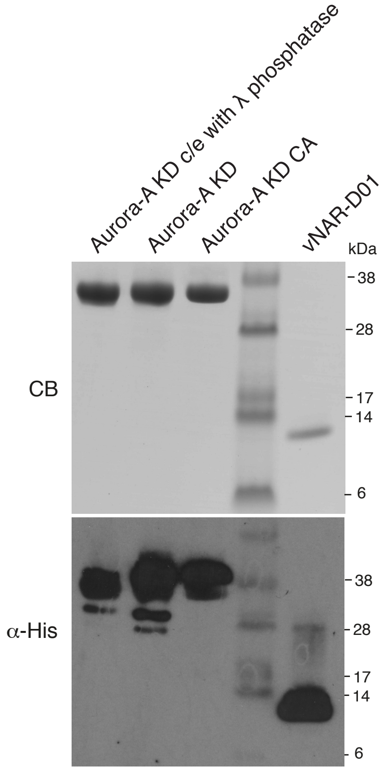

Figure S1. Characterisation of vNAR-D01.
(A) Sequence of vNAR-D01. The CDR1 region is shown in blue, the HV2 sequence is shown in orange and the CDR3 region is in red. (B) Far Western of Aurora-A KD constructs probed with vNAR-D01 and resolved by anti-His Western. Proteins were analysed by SDS-PAGE (top panel). CB, Coomassie blue. Binding of vNAR-D01 was confirmed by Far Western blotting (bottom panel). The vNAR-D01 was loaded as a positive control for the anti-His Western.

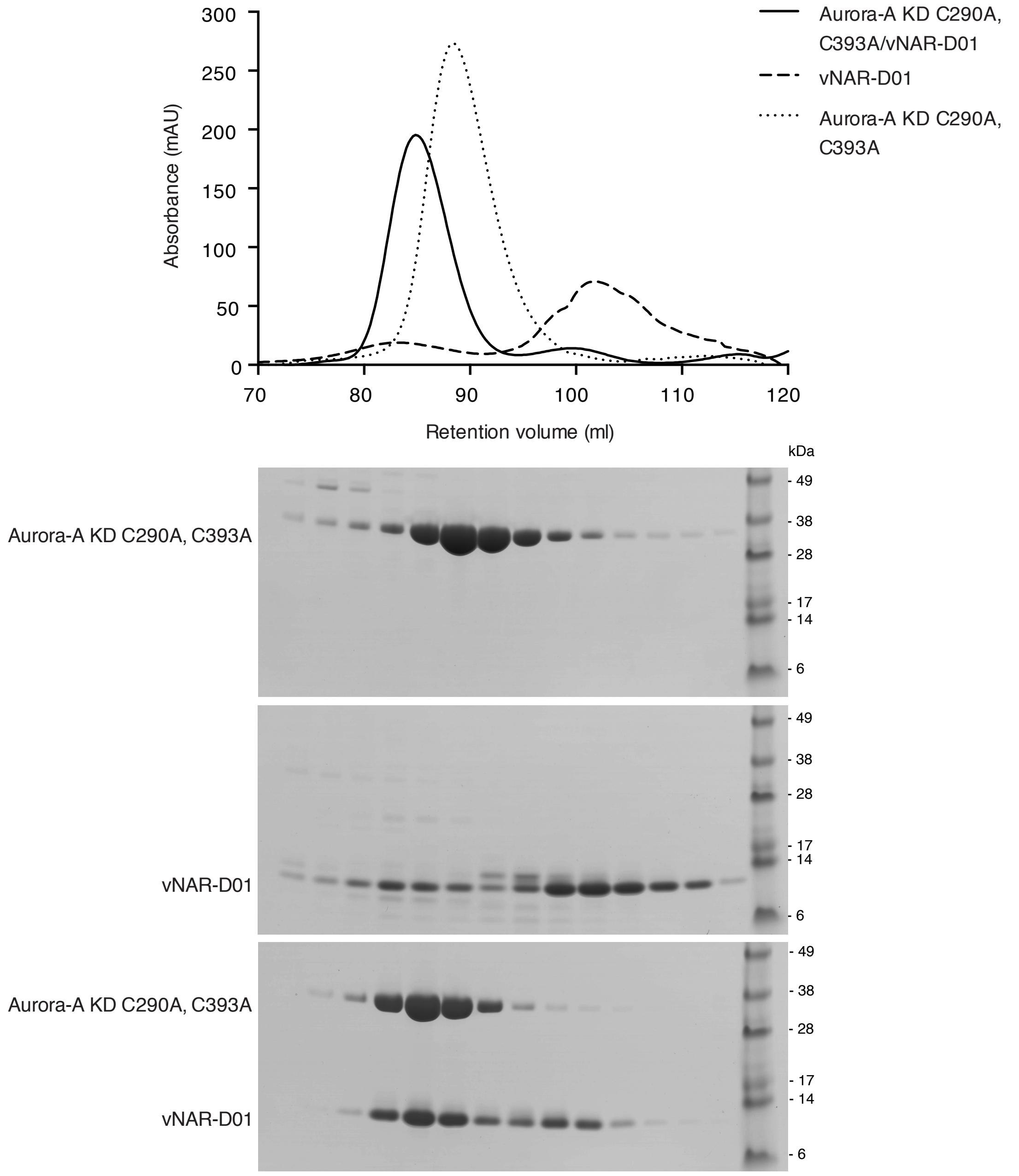

Figure S2. Characterization of the Aurora-A/vNAR-D01 complex by SEC.
SEC of Aurora-A KD CA and vNAR-D01 alone and in complex. The proteins were subject to SEC on a HiLoad Superdex S200 16/60 column and elution profiles recorded (top panel). Fractions across the elution volume were subject to SDS-PAGE analysis (below).

A

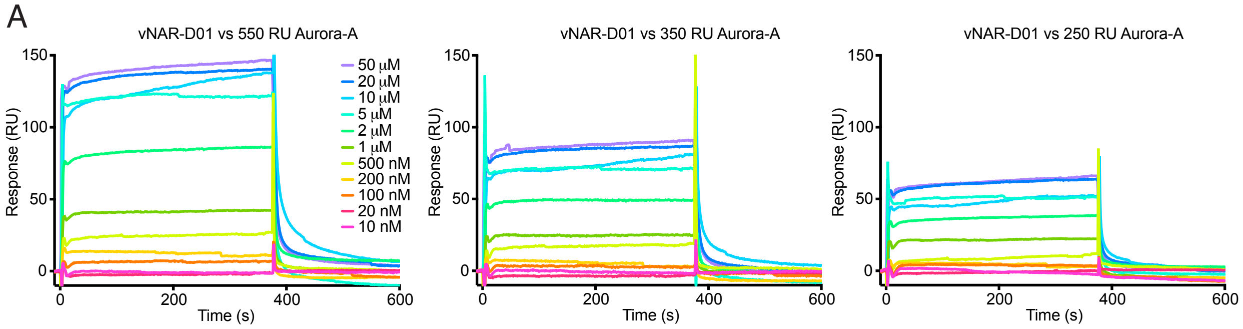

[Aurora-A] (RU)	KD (µM)
550	2.0 ± 0.2
350	2.2 ± 0.3
250	2.0 ± 0.3

B

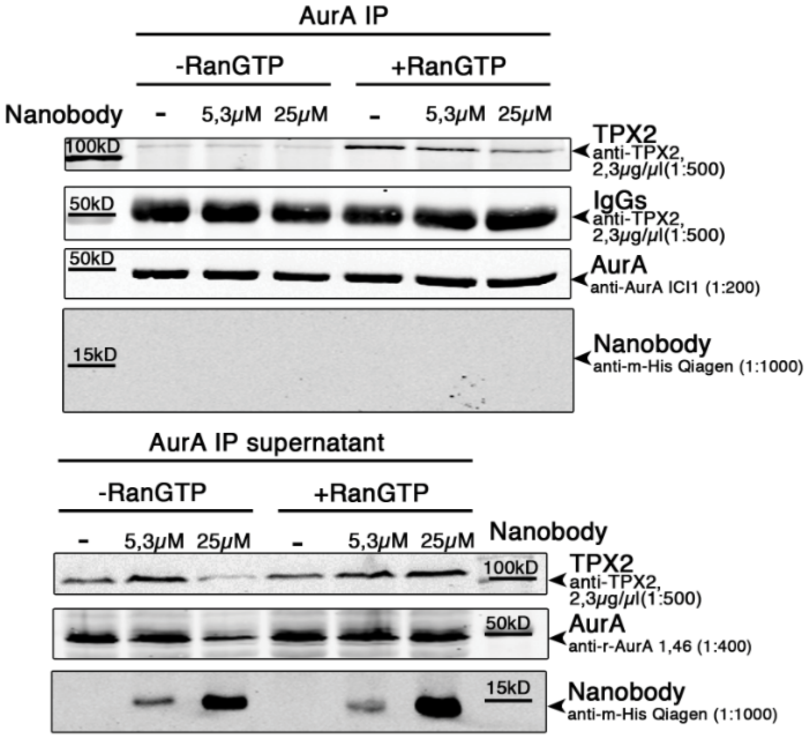

Figure S3. Characterization of the Aurora-A/vNAR-D01 interaction.
(A) SPR experiments were performed to measure the binding affinity between Aurora-A KD-Avi and vNAR-D01. The sensorgrams recorded are shown (above). Binding affinities measured are stated in the Table (below). (B) Aurora-A IP assays performed in *Xenopus* egg extracts in the presence of vNAR-D01 (labelled Nanobody). TPX2 efficiently co-precipitates with Aurora-A only in the presence of RanGTP. However, vNAR-D01 did not co-precipitate with Aurora-A. The binding site of vNAR-D01 is conserved in *Xenopus* Aurora-A, with the exception of a conservative substitution (R251K), so the most likely explanation for the lack of binding is that the vNAR domain has insufficient potency to compete with TPX2 in a complex cell extract.

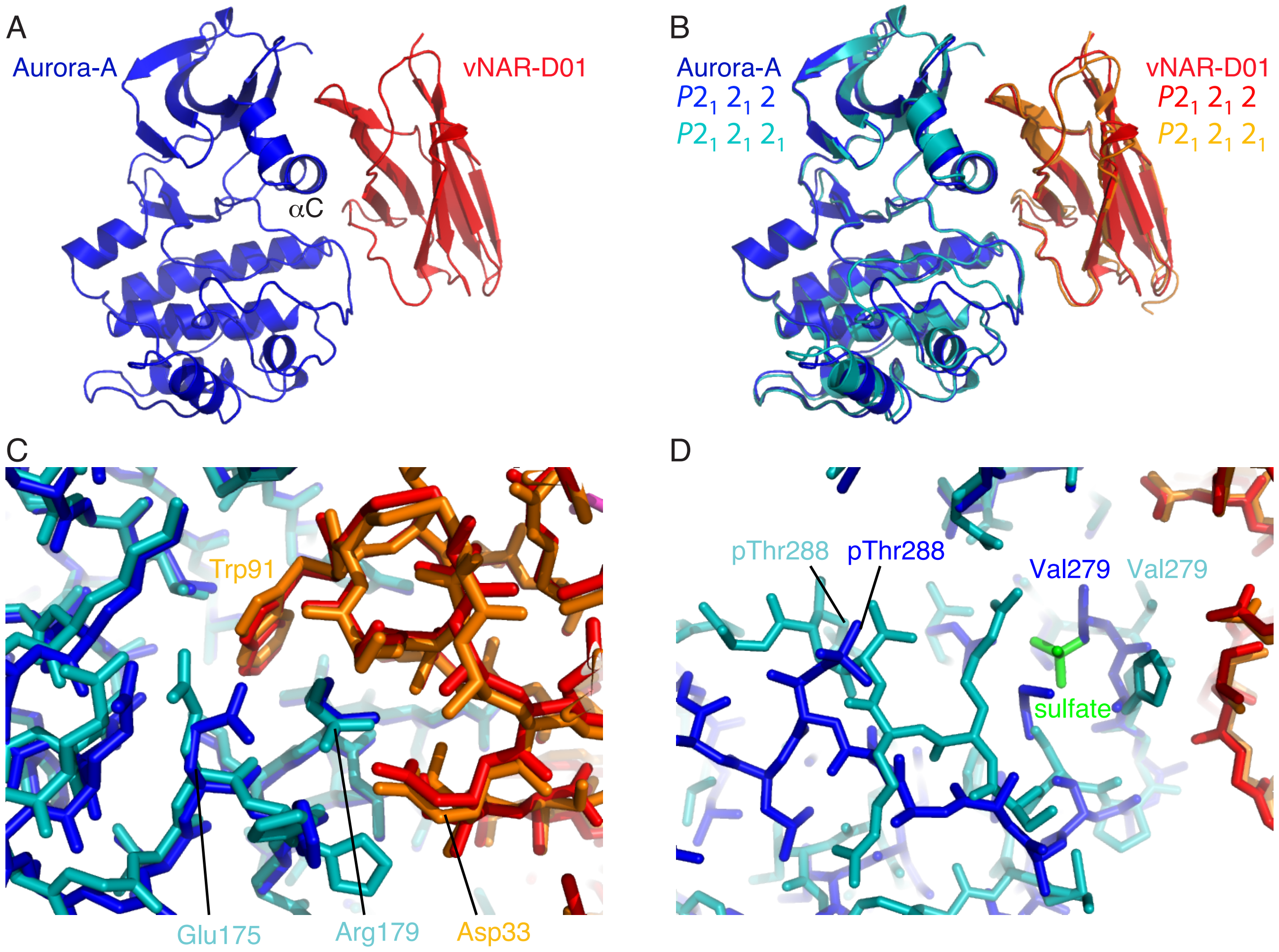

Figure S4. Comparison of two crystal forms of Aurora-A KD CA/vNAR-D01
(A) Cartoon representation of the complex structure (crystal form 2). (B) Superposition of the two crystal forms. (C) Magnified view of the superposed structures in the vicinity of vNAR-D01 Trp91 shows the overall similarity of the key binding interface. (D) Magnified view of superposed structures in the vicinity of the Aurora-A residue pThr288 shows a divergent conformation of the activation loop between residues 282 and 295. This is most likely due to differences in the crystal packing contacts.

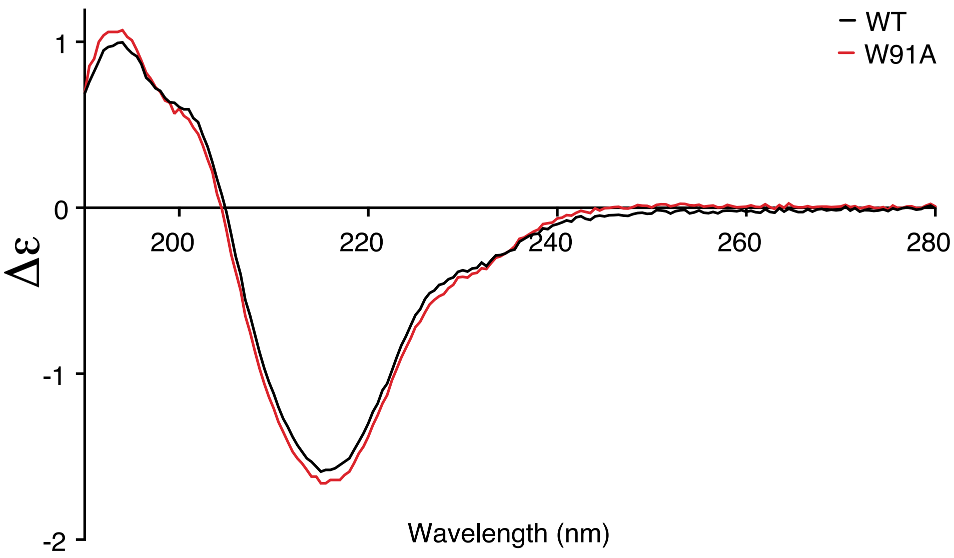

Figure S5. CD spectra of wild-type vNAR-D01 and the point mutant, W91A.

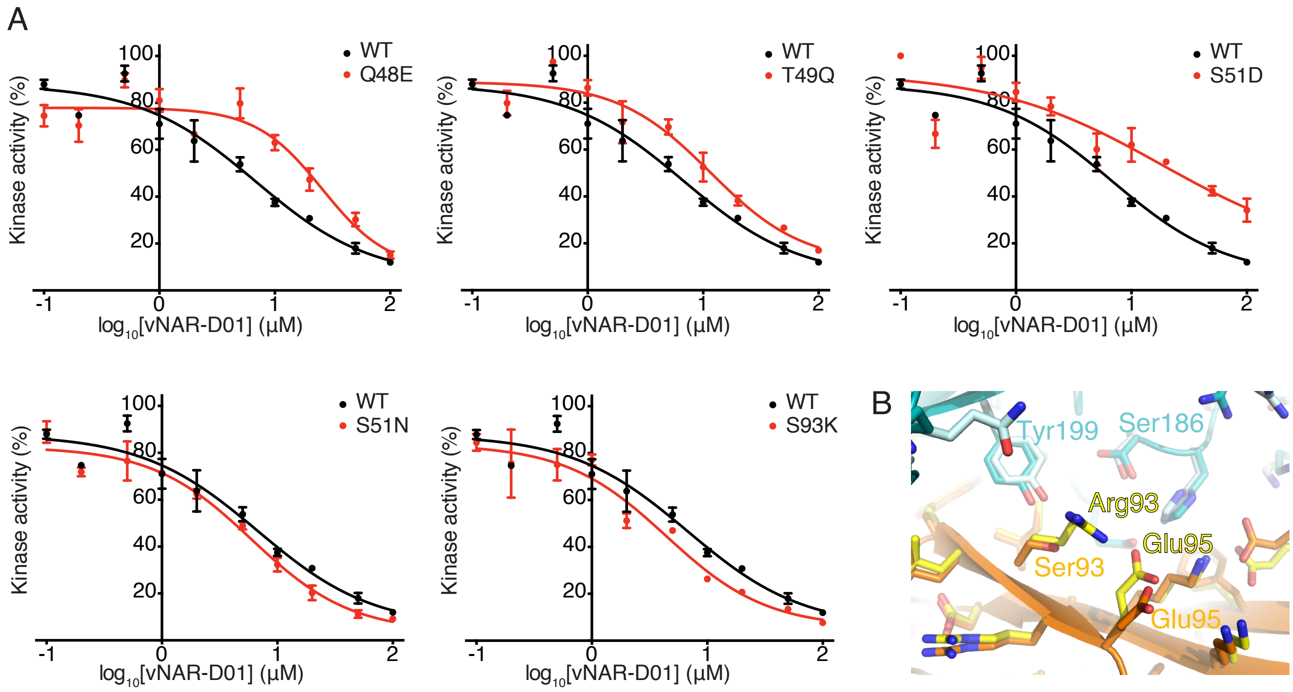

Figure S6. Characterization of vNAR-D01 mutants by kinase activity assay and crystallography. (A) I*n vitro* kinase activity curves of Aurora-A KD in the presence of WT and mutant vNAR-D01 proteins. The kinase activity of Aurora-A KD was measured by the incorporation of radioisotope into the generic kinase substrate, MBP by scintillation counting in the presence of 0.1, 0,2 0.5, 1, 2, 5, 10, 20, 50 and 100 µM vNAR-D01. Data were normalized to % kinase activity using the Aurora-A KD only reaction as 100% and plotted against vNAR-D01 concentration. Data were fitted to a log(inhibitor) vs. response – variable slope in Prism6 (Graphpad) to calculate IC50s (Figure 1E). (B) Crystal structure of Aurora-A KD CA/vNAR-D01 S93R (coloured white and yellow, respectively) superimposed onto the structure of Aurora-A KD CA/vNAR-D01 (coloured cyan and orange, respectively)

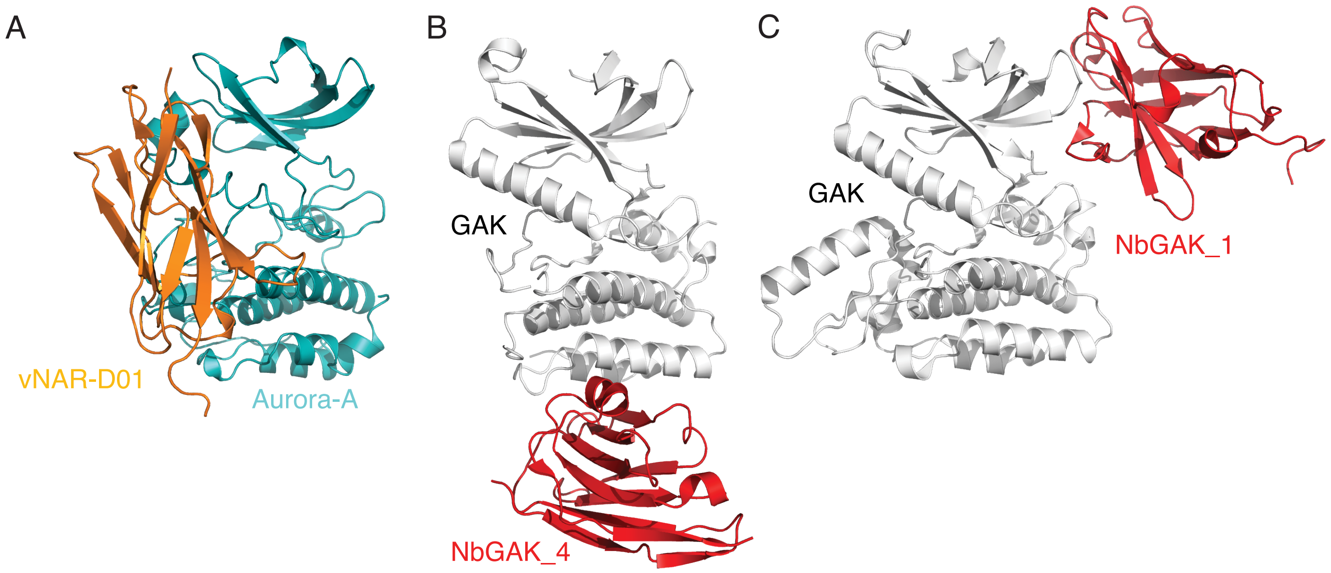

Figure S7. Crystal structures of kinase/single variable domain complexes. (A) Overview of the Aurora-A/vNAR-D01 complex. (B) NbGAK_4 binds to the C-lobe of GAK (PDB 4C58). (C) NbGAK_1 binds to the N-lobe of GAK (PDB 4C57).

	Aurora-A KD CA /vNAR-D01 Crystal 1	Aurora-A KD CA /vNAR- D01 Crystal 2	Aurora-A KD CA/ vNAR- D01 S93R
**Data collection**			
Space group	$P2_1\ 2_1\ 2_1$	$P2_1\ 2_1\ 2$	$P2_1\ 2_1\ 2$
Cell dimensions			
a, *b*, *c* (Å)	68.14, 84.99, 88.87	88.70, 109.72, 45.76	91.23, 111.12, 41.62
α, β, γ (°)	90.00, 90.00, 90.00	90.00, 90.00, 90.00	90.00, 90.00, 90.00
Resolution range (Å)	45.62-1.67 (1.71 –1.67)*	46.66-1.79 (1.84-1.79)*	55.56-1.68 (1.72-1.68)*
R_{merge} (%)	4.6 (146)	5.7 (161)	4.3 (149)
R_{pim} (%)	2.1 (65.3)	2.6 (72.2)	2.0 (69.8)
$I / \sigma I$	19.0 (1.3)	17.7 (1.2)	19.6 (1.2)
Completeness (%)	99.4 (99.2)	97.8 (91.4)	99.8 (99.9)
Redundancy	6.7 (6.8)	6.7 (6.7)	6.5 (6.3)
**Refinement**			
Resolution (Å)	44.46-1.67	46.66-1.79	55.56-1.68
No. reflections	60047	41890	48994
R_{work} / R_{free}	19.10/22.33	19.02/23.51	19.93/23.10
No. atoms			
Protein	3058	2889	2795
Water	348	280	197
Hetero	82	77	49
Mean *B*-factors			
Protein	33.53	37.28	35.47
Hetero	54.66	50.32	47.70
Water	46.15	46.63	42.77
Wilson *B*-factor	27.91	29.84	29.36
r.m.s. deviations			
bond lengths (Å)	0.007	0.007	0.008
bond angles (°)	1.160	1.227	1.042
**MolProbity analysis**			
All-atom clash-score	5.31	4.12	1.61
Rotamers outliers (%)	0.93	1.01	0.34
Ramachandran outliers (%)	0	0.27	0
Ramachandran favoured (%)	97.66	96.49	97.73
MolProbity score	1.36	1.43	0.97

*Values in parentheses are for highest-resolution shell.

Table S1. Data collection and refinement statistics.
